# Outcome in Critically Ill Dogs and Dogs With Acute Kidney Injury Based on Neutrophil Gelatinase‐Associated Lipocalin and Tissue Inhibitor of Metalloproteinase‐2

**DOI:** 10.1111/jvim.70024

**Published:** 2025-02-26

**Authors:** Elisabeth Dorn, Ann Biscop, Nausikaa Devriendt, Donatienne Castelain, Kristel Demeyere, Emmelie Stock, Evelyne Meyer, Dominique Paepe

**Affiliations:** ^1^ Small Animal Department Ghent University Merelbeke Belgium; ^2^ Department of Veterinary and Biosciences Ghent University Merelbeke Belgium; ^3^ Department of Morphology, Imaging, Orthopedics, Rehabilitation and Nutrition Ghent University Merelbeke Belgium

**Keywords:** canine, cell cycle arrest biomarker, early diagnosis, kidney disease, renal biomarker

## Abstract

**Background:**

Neutrophil gelatinase‐associated lipocalin (NGAL) and tissue inhibitor of metalloproteinase‐2 (TIMP‐2) have potential as early biomarkers for acute kidney injury (AKI) in dogs.

**Objectives:**

Assess whether NGAL and TIMP‐2 at admission (T0) and 24 h later (T1) identify survival in critically ill (CI) and AKI dogs, development of hospital‐acquired AKI in CI dogs, and development of chronic kidney disease (CKD) in AKI dogs after 3 months.

**Animals:**

Sixty‐two client‐owned dogs: 10 healthy, 24 with AKI, and 28 CI.

**Methods:**

Prospective study with blood and urine samples collected at T0, T1, and up to 1 week in CI dogs, 1 month in healthy dogs, and 3 months in AKI dogs. Serum and urinary NGAL (sNGAL; uNGAL) and urinary TIMP‐2 (uTIMP‐2) were measured using validated ELISA kits.

**Results:**

Dogs with AKI that did not survive had significantly higher uNGAL concentrations and u/sNGAL ratios at T0 compared with survivors (*p* = 0.05, *n* = 23; and *p* = 0.03, *n* = 21, respectively). In CI dogs, sNGAL was significantly higher in non‐survivors at T0 and T1 compared with survivors (*p* = 0.02, *n* = 26; and *p* = 0.003, *n* = 26, respectively). At T0, normalized urinary tissue inhibitor of metalloproteinase‐2 (u_norm_TIMP‐2) was significantly higher in non‐survivor CI dogs compared with survivors (*p* = 0.04, *n* = 25). No significant differences were found for the other variables.

**Conclusions and Clinical Relevance:**

In AKI dogs, uNGAL and u/sNGAL at T0, and in CI dogs, sNGAL at T0 and T1 and u_norm_TIMP‐2 at T0, were potential predictors of survival.

**Abbreviations**
AKI: acute kidney injury
CI: critically ill
CKD: chronic kidney disease
FeNGAL: fractional excretion of neutrophil‐gelatinase associated lipocalin
IL: interleukin
IRIS: International Renal Interest Society
LOD: limit of detection
LOQ: limit of quantification
NGAL: neutrophil‐gelatinase associated lipocalin
PCR: polymerase chain reaction
sCr: serum creatinine
SDMA: symmetric dimethylarginine
sNGAL: serum neutrophil‐gelatinase associated lipocalin
sUrea: serum urea
T0: time point 0 (at initial presentation)
T1: time point 1 (after 24 h)
T2: time point 2 (after 48 h)
T3: time point 3 (after 1 week)
T4: time point 4 (after 1 month)
T5: time point 5 (after 3 months)
u/sNGAL: urinary to serum neutrophil‐gelatinase associated lipocalin
uNGAL: urinary neutrophil‐gelatinase associated lipocalin
u_norm_/sNGAL: urinary to serum neutrophil‐gelatinase associated lipocalin with normalized NGAL
u_norm_NGAL: normalized urinary neutrophil‐gelatinase associated lipocalin
u_norm_TIMP‐2: normalized urinary tissue inhibitor of metalloproteinase‐2
UPC: urinary protein:creatinine ratio
USG: urine specific gravity
uTIMP‐2: urinary tissue inhibitor of metalloproteinase‐2

## 1 Introduction

Acute kidney injury (AKI) is characterized by sudden damage to the kidney. It represents a continuum of kidney injury from mild, clinically inapparent, nephron loss to severe acute kidney failure [1, 2]. The most common causes include ischemia, inflammation, nephrotoxin exposure, and infectious diseases [3, 4, 5]. However, AKI can also emerge as a potentially life‐threatening complication of various medical conditions in hospitalized dogs, referred to as hospital‐acquired AKI. The mortality rate of dogs with community‐acquired AKI ranges from 34% to 45%, whereas values from 44% to 86% have been reported for hospital acquired AKI [4, 6, 7]. Any acute injury to the glomerular or tubulo interstitial regions can trigger the onset of chronic kidney disease (CKD) [8, 9].

Unfortunately, early diagnosis of AKI, in particular identification of non‐azotemic AKI or International Renal Interest Society (IRIS) grade 1 AKI, remains challenging. Nonetheless, early detection is imperative for timely intervention and improved clinical outcomes [10].

The damage biomarker neutrophil‐gelatinase associated lipocalin (NGAL) is a low molecular weight protein that is substantially upregulated in response to ischemic kidney injury [11]. It has been extensively studied as a biomarker for AKI because of its rapid release from renal tubular cells during the early phases of kidney injury [12]. It was shown that NGAL is an early and sensitive indicator of AKI, with concentrations increasing in urine and blood within hours after kidney damage [13]. Its potential for early diagnosis and differentiation of AKI from other kidney diseases has been demonstrated in both human and veterinary medicine [13, 14, 15]. Furthermore, it also has been explored as a potential predictor of recovery [16], with changes in its concentration correlating with the healing process. Its expression is triggered by tubular stress or injury, making it a key marker for both the onset and progression of AKI [17].

The urinary cell cycle arrest biomarker, urinary tissue inhibitor of metalloproteinase‐2 (uTIMP‐2), is a stress biomarker that recently has shown promise in distinguishing AKI from other conditions in dogs [18]. It is an inflammatory marker that plays a key role in various physiological and pathological processes, particularly in kidney injury and repair [19]. Although TIMP‐2 is expressed at high concentrations in several tissue types, its primary expression within the nephron is within the collecting ducts [20]. Recent data indicate that uTIMP‐2, along with other biomarkers such as NGAL, can help in the early diagnosis of AKI by detecting kidney stress before clinically relevant functional decline, allowing for better differentiation between AKI and other diseases [18].

Data predicting the outcome of AKI dogs and critically ill (CI) dogs based on renal biomarkers is lacking. Therefore, our prospective, longitudinal study aimed to evaluate the association of several biomarkers in AKI and CI dogs with different outcomes. Specifically, we aimed to assess whether serum and urinary NGAL (sNGAL and uNGAL), uTIMP‐2, and routine serum and urine biomarkers (serum urea [sUrea], serum creatinine [sCr], and symmetric dimethylarginine [SDMA] concentrations, urine specific gravity [USG], and urinary protein‐to‐creatinine ratio [UPC]), measured at admission (T0) and 24 h later (T1), could identify the following: (1) surviving versus nonsurving dogs with AKI or CI dogs, including overall survival, short‐term survival, and long‐term survival in both AKI and CI dogs; (2) which CI dogs developed AKI during hospitalization; and (3) which AKI dogs subsequently developed CKD 3 months after admission.

## 2 Material and Methods

Our prospective observational study was carried out at the Small Animal Clinic, Ghent University, and received approval from the Ethical Committee of the Faculty of Veterinary Medicine, Ghent University, Belgium, and the deontological committee of the Belgian Federal Agency for the Safety of the Food Chain (EC 2020/067; approval date: November 19, 2020, Deontological committee: 2018‐86).

### 2.1 Study Cohort

After obtaining signed informed consent from their owners, healthy, AKI, and CI dogs were enrolled in the study as previously described [18]. Dogs with AKI and CI dogs were included upon admission, provided they met the inclusion criteria and sampling was feasible. All dogs underwent general physical examination, non‐invasive blood pressure measurement using Doppler, CBC, serum biochemistry profile, urinalysis (including urine culture), and abdominal ultrasonography to determine their eligibility for the study. Additional diagnostic tests were conducted for AKI and CI dogs at the discretion of the attending clinician. Dogs were excluded if their body weight was < 2 kg, if they had severe thrombocytopenia (< 20 000/μL), coagulation disorders, or severe anemia (hematocrit < 13.0%).

Dogs were classified as healthy based on their medical history and the absence of clinically relevant abnormalities detected during physical examination, blood analyses, urinalysis, and diagnostic imaging (abdominal ultrasonography and thoracic radiography). The dogs had not received drugs for at least 2 months before inclusion, except for preventive treatments.

Dogs were identified as having AKI if they had an acute onset of clinical signs (< 14 days) and met at least two of the following criteria: (1) acute onset of renal azotemia with sCr concentrations > 1.8 mg/dL (upper reference limit determined by the in‐house IDEXX Catalyst Dx chemistry analyzer, IDEXX Laboratories Inc., Westbrook, Maine) that persisted for a minimum of 24 h after correction of prerenal factors; (2) indicators of acute tubular injury on urinalysis, such as glucosuria without hyperglycemia or the presence of urinary casts; (3) imaging results indicative of AKI, including normal or increased renal size, perirenal effusion, or perirenal steatitis; (4) a progressive, non‐azotemic increase in sCr (> 0.3 mg/dL) within 48 h; and (5) persistent oliguria or anuria for more than 6 h after dehydration correction. Dogs were excluded if they had a history or ultrasonographic evidence of CKD or had been treated with drugs that influence kidney perfusion (e.g., angiotensin‐converting enzyme inhibitors, angiotensin‐receptor blockers, dopamine, calcium channel blockers) within 10 days before inclusion. All AKI dogs were tested for leptospiral infection using a serum microagglutination test and real‐time quantitative polymerase chain reaction (PCR) on urine samples.

Inclusion in the CI group necessitated admission to the emergency department for a potentially life‐threatening illness and attainment of an acute patient physiologic and laboratory evaluation (APPLE) fast score of ≥ 18. Dogs presenting with clinicopathological or ultrasonographic indications of AKI (as previously defined), CKD, or post‐renal azotemia were excluded from the study.

Nonsurvivors were defined as animals that died or were euthanized because of clinical deterioration during the study period. Dogs that were euthanized mainly for financial reasons were excluded from the study.

### 2.2 Sample Collection

Blood and urine samples were collected within 4 h of admission (T0). Additional samples were collected after rehydration at 24 h (T1), 48 h (T2), 1 week (T3), 1 month (T4; only in healthy and AKI), and 3 months (T5; only in AKI dogs) after admission. Except for AKI and CI dogs at T0 and T1, dogs were fasted before blood collection. Blood (10 mL) was drawn by standard venipuncture from the jugular vein using a 21 G needle and divided into serum, heparin, and EDTA tubes. Simultaneously, urine samples (10 mL) were obtained. At T0, blood and urine samples ideally were collected simultaneously and before IV fluid administration. However, in cases such as anuric dogs or clinically unstable dogs, urine sampling was delayed for up to 2 h after initial stabilization and during fluid therapy. At T1 and T2 all dogs, and at T3 some AKI and CI dogs were receiving fluid therapy at the time of sampling.

Subsequently, serum and urine samples were sent to an external laboratory within 48 h for routine kidney function assessment, including SDMA, and urinalysis. Any remaining serum and urine supernatants were aliquoted into 300 μL portions and stored at −80°C within 24 h for up to 2 years before biomarker analyses in batch.

### 2.3 Routine Assessment of Kidney Function

The following variables were assessed (IDEXX Bioanalytics, Kornwestheim, Germany) in serum: sCr, sUrea, SDMA, potassium, sodium, chloride, inorganic phosphate, total calcium, albumin, and total protein. Serum creatinine concentration was measured using Jaffe's kinetic method without deproteinization (manufacturer Beckman Coulter). Quantification of SDMA concentration was performed using the validated immunoassay IDEXX SDMA Test (IDEXX Laboratories Inc., Westbrook, Maine). Concentrations > 14.0 μg/dL were considered increased. Urinalysis was performed by Sonic Healthcare Benelux (Belgium) and consisted of USG, dipstick analysis, urinary protein creatinine ratio (UPC), and urine culture. Additionally, microscopic sediment analysis was performed on‐site within 60 min after collection. For UPC determination, urinary protein concentration was measured by turbidimetry and urinary creatinine concentration (uCr) by spectrophotometry (using Jaffe's kinetic method) using Cobas c702 (Roche Diagnostics).

### 2.4 Serum and Urinary NGAL

Aliquots of serum and urine were thawed for 2 h at room temperature before dilution and NGAL analyses. Both sNGAL and uNGAL were measured using a commercially available, NGAL ELISA kit designed for dogs (Dog NGAL ELISA Kit, Bioporto, Hellerup, Denmark), following the manufacturer's instructions as recently described [18]. This assay detects concentrations ranging from 0.6 to 400.0 pg/mL. Serum and urine samples were diluted based on the UPC [15, 18]. The minimum detectable dose (limit of detection, LOD) was defined as 0.6 pg/mL according to the manual, and the limit of quantification (LOQ) was defined as 9.6 pg/mL in correlation with the standard recovery [14]. The results were multiplied with the dilution factor before further analysis. The results of sNGAL and uNGAL are presented as absolute concentrations and uNGAL subsequently was normalized to uCr and expressed as u_norm_NGAL.

The following additional variables were calculated:

1. Urinary to serum NGAL ratio with uNGAL (u/sNGAL) = uNGAL/sNGAL.
2. Urinary to serum NGAL ratio with u_norm_NGAL (u_norm_/sNGAL) = [uNGAL/uCr]/sNGAL.
3. Fractional excretion of NGAL (FeNGAL) = [uNGAL/sNGAL]/[uCr/sCr] × 100.

### 2.5 Urinary TIMP‐2

Before measurement, urine aliquots were thawed for 2 h at room temperature. Concentrations of uTIMP‐2 were measured according to the manufacturer's procedure using the canine TIMP‐2 ELISA (Abcam, Cambridge, UK) kit after in‐house validation (intra‐assay coefficient of variation, sensitivity, and matrix interference [spiking, linearity]) as recently described [18]. The results were multiplied with the dilution factor (×2) before further analysis. The LOD of TIMP‐2 was determined to be 5.1 pg/mL [18]. The limit of quantification (LOQ) was defined as 20.0 pg/mL, which is the lowest standard concentration. Both absolute and normalized (uTIMP‐2/uCr) TIMP‐2 results were examined.

### 2.6 Statistical Analyses

Statistical analyses were performed using a statistical software package (SPSS Statistics 29, IBM, Armonk, USA). The normality of variables was assessed using Shapiro–Wilk tests. Differences in variables at T0–T3 between groups were assessed by one‐way analysis of variance (ANOVA) for parametric data, whereas Kruskal–Wallis tests were used for non‐parametric data. At T4, differences between groups were evaluated using Mann–Whitney *U* tests. Friedman's 2‐way ANOVA tests were performed to analyze changes over time. In each group, short‐term evolution of different variables over time (T0–T2) was assessed separately from long‐term evolution (T0 until the end of the study: T3 for CI, T4 for healthy, and T5 for AKI dogs). Mann–Whitney *U* tests were used to evaluate if values at T0 and T1 could predict survival in CI dogs and dogs with AKI.

Development of AKI in CI dogs was diagnosed when at least one of the following criteria was present: increase in sCr ≥ 1.6 mg/dL, progressive nonazotemic increase in sCr ≥ 0.3 mg/dL within 48 h, oliguria or anuria over 6 h, new evidence of AKI on abdominal ultrasonography such as increasing kidney size, perirenal effusion or perirenal steatitis. Further grading (Grades I–V) was performed according to the International Renal Interest Society (IRIS) Guidelines [2]. Mann–Whitney *U* tests were used to evaluate if values at T0 and T1 differed in CI dogs that did develop AKI compared to those that did not develop AKI until T3.

Development of CKD in dogs initially presented for AKI was assessed using sCr or SDMA concentration, urinalysis, and abdominal ultrasonography 3 months after initial presentation (T5) in a hydrated, stable patient. Grade 1 CKD was diagnosed in dogs with normal sCr (< 1.4 mg/dL) or normal or mildly increased SDMA (< 18.0 μg/dL), and other kidney abnormalities present (such as abnormal renal imaging findings, isosthenuria, proteinuria, or glucosuria of renal origin). According to IRIS guidelines, dogs with sCr > 1.4 mg/dL or SDMA > 18.0 μg/dL were staged as having CKD and substaged based on proteinuria and systemic blood pressure [21]. Mann–Whitney *U* tests were used to evaluate if results at T0 and T1 differed in AKI dogs that did develop CKD compared to those that did not develop CKD at T5.

Whenever multiple comparison tests were performed and statistical differences were present, Bonferroni correction was applied. Results were considered significant if (adjusted) *p* was ≤ 0.05.

## 3 Results

### 3.1 Study Cohort and Routine Assessment of Kidney Function

The study sample consisted of 10 healthy dogs, 24 dogs with AKI, and 28 CI dogs of various breeds. Relevant clinicopathological data of the three groups are presented in Table 1. The clinicopathological data during follow‐up are shown in Table S1. Because of factors such as insufficient sample size, anuria, and death, not all measurements of all animals were available at every time point. Both at baseline and at different time points, differences in variables were found among the groups (Table 1 and Table S1).

**TABLE 1:** Clinical and clinicopathological data for all dog groups at initial presentation (T0).

Variable (unit) [reference interval]	Healthy dogs	AKI dogs	CI dogs
Age (months)	55.1 ± 23.5	86.2 ± 46.4	74.1 ± 41.4
	*n* = 10	*n* = 24	*n* = 28
Body weight (kg)	19.0 ± 11.7	25.7 ± 12.3	22.3 ± 13.0
	*n* = 10	*n* = 24	*n* = 28
Non‐invasive systolic blood pressure (mmHg)	140.3 ± 12.0	135.8 ± 34.1	122.3 ± 22.2
	*n* = 10	*n* = 23	*n* = 22
sCr (mg/dL) [0.5–1.8]	1.0 (0.6–1.6)^b^	3.9 (1.4–17.6)^a^, ^c^	0.8 (0.3–3.7)^b^
	*n* = 10	*n* = 24	*n* = 28
sUrea (mmol/L) [3.2–10.3]	4.8 (2.1–11.8)^b^	25.5 (5.3–98.3)^a^, ^c^	6.1 (1.9–33.8)^b^
	*n* = 10	*n* = 24	*n* = 28
SDMA (μg/dL) [0.0–14.0]	12.0 (7.0–14.0)^b^	37.0 (12.0–> 100.0)^a^, ^c^	12.5 (6.0–46.0)^b^
	*n* = 10	*n* = 23	*n* = 28
Potassium (mmol/L) [3.9–5.8]	4.2 (4.0–4.6)	4.3 (3.6–6.6)	4.2 (3.2–6.0)
	*n* = 10	*n* = 24	*n* = 28
Sodium (mmol/L) [142.0–153.0]	146.9 ± 1.3	143.8 ± 7.1	145.4 ± 5.0
	*n* = 10	*n* = 24	*n* = 28
Chloride (mmol/L) [106.0–120.0]	113.0 (109.0–117.0)	106.5 (82.0–119.0)	112.0 (83.0–119.0)
	*n* = 10	*n* = 24	*n* = 28
Phosphorus (mmol/L) [0.9–1.7]	1.1 (0.7–1.4)^b^, ^c^	2.5 (0.9–8.0)^a^, ^c^	1.6 (1.1–4.2)^b^, ^c^
	*n* = 10	*n* = 24	*n* = 28
Calcium (mmol/L) [2.1–2.9]	2.5 (2.2–2.7)^c^	2.5 (1.3–2.8)^c^	2.3 (1.9–2.6)^a^, ^b^
	*n* = 10	*n* = 24	*n* = 28
Albumin (g/L) [28.0–43.0]	31.7 ± 4.0^b^, ^c^	24.8 ± 5.7^a^	23.3 ± 5.5^a^
	*n* = 10	*n* = 24	*n* = 28
Total protein (g/L) [54.0–76.0]	64.3 ± 7.6^c^	55.6 ± 12.0	49.5 ± 12.8^a^
	*n* = 10	*n* = 24	*n* = 28
USG [1.015–1.045]	1.035 (1.022–1.050)^b^	1.014 (1.005–1.024)^a^, ^c^	1.026 (1.004–1.050)^b^
	*n* = 10	*n* = 24	*n* = 25
UPC [< 0.50]	0.07 (0.05–0.11)^b^, ^c^	0.89 (0.17–70.36)^a^	0.40 (0.01–4.75)^a^
	*n* = 10	*n* = 24	*n* = 25
Glucosuria in absence of hyperglycemia	1/9	13/24	9/25

*Note:* Results are expressed as mean ± standard deviation for parametric data and median (range) for non‐parametric data.

Abbreviations: AKI, acute kidney injury; CI, critically ill; sCr, serum creatinine; SDMA, symmetric dimethylarginine; sUrea, serum urea; UPC, urinary protein:creatinine ratio; USG, urine specific gravity.

^a^ Significant difference compared to healthy dogs.

^b^ Significant difference compared to AKI dogs.

^c^ Significant difference compared to CI dogs.

One healthy dog had glucosuria at the time of sampling, which was not detected in both previous and follow‐up urinalyses. Furthermore, one healthy dog had mildly increased sUrea with a normal sCr (0.9 mg/dL) and concentrated urine (USG 1.050).

According to the IRIS grading system, the AKI group consisted of one dog with IRIS grade 1 (4.1%), five dogs with IRIS grade 2 (20.8%), seven dogs with IRIS grade 3 (29.2%), six dogs with IRIS grade 4 (25%), and five dogs with IRIS grade 5 (20.8%). Diagnoses or underlying diseases in the AKI group included leptospirosis (*n* = 5), hypercalcemia (*n* = 1), non‐steroidal anti‐inflammatory drug administration (*n* = 1), pyelonephritis (*n* = 1), diabetic ketoacidosis (*n* = 1), acute hemorrhagic diarrhea syndrome (*n* = 1), septic peritonitis caused by pyometra (*n* = 1), and rhabdomyolysis (*n* = 1). One dog was diagnosed with leptospirosis, obstructive uroliths (cystine), and bacterial cystitis. Two dogs were strongly suspected of leptospirosis but it was not confirmed by PCR or serology. In nine dogs, the cause of AKI remained unknown.

The CI group consisted of six dogs diagnosed with acute hemorrhagic diarrhea syndrome, four dogs with septic peritonitis, four dogs with immune‐mediated disease (two hemolytic anemia, one panniculitis, and one polyarthritis), two dogs with sepsis, two dogs with acute hepatopathy, two dogs with hemoabdomen, and one dog each with aspiration pneumonia, amanita intoxication with severe hepatopathy, cholecystitis, diabetic ketoacidosis, lung lobe torsion, parvovirosis, uroabdomen, and one with subobstructive choledocholithiasis and focal lymphadenitis. Three CI dogs had mildly increased sCr (2.0, 2.3, and 3.7 mg/dL) at presentation which normalized after 24 h and was indicative of prerenal azotemia, and hence were not excluded from the study.

### 3.2 Changes Over Time in Serum and Urinary Biomarkers Within Groups

Short‐term evolution over time (from T0 to 24 h later at T1, or 48 h later at T2) resulted in significant changes in sCr (*p* = 0.02, *n* = 10) in healthy dogs. However, no significant changes were found after multiple comparison tests. Furthermore, in healthy dogs, uNGAL was significantly lower at T2 versus T0 (*p* = 0.03, *n* = 5), and u_norm_NGAL was significantly lower at T1 versus T0 (*p* = 0.01, *n* = 5). Additionally, uTIMP‐2 (*p* = 0.04, *n* = 10) had significant changes for short term evolution over time. However, no significant changes were obtained after multiple comparison tests.

In AKI dogs, sCr, sUrea, and SDMA were significantly lower at T1 versus T0 (*p* < 0.001, *p* = 0.50, and *p* = 0.001, respectively, all *n* = 21) and at T2 versus T0 (*p* < 0.001, *p* < 0.001, and *p* = 0.03, respectively, all *n* = 21). In AKI dogs, UPC, uNGAL, and u_norm_/sNGAL differed significantly (*p* = 0.03, *n* = 18; *p* = 0.03, *n* = 14; and *p* = 0.050, *n* = 13, respectively). However, again, no significant changes were obtained after multiple comparison tests among T0, T1, and T2.

In CI dogs, sCr was significantly lower at T1 and T2 versus T0 (both *p* < 0.001, *n* = 26), and sUrea was significantly lower at T1 versus T0 (*p* = 0.01, *n* = 26).

With long‐term evolution over time, healthy dogs had significantly lower sCr (*p* = 0.04, *n* = 10). However, no significant differences were present after multiple comparison tests of T0–T4. Similarly, uNGAL (*p* = 0.004, *n* = 5) was significantly lower, but again no significant differences were present after multiple comparison tests of T0–T4. In contrast, u_norm_NGAL at T3 versus T0 (*p* = 0.02, *n* = 4) was significantly lower.

**FIGURE 1:**
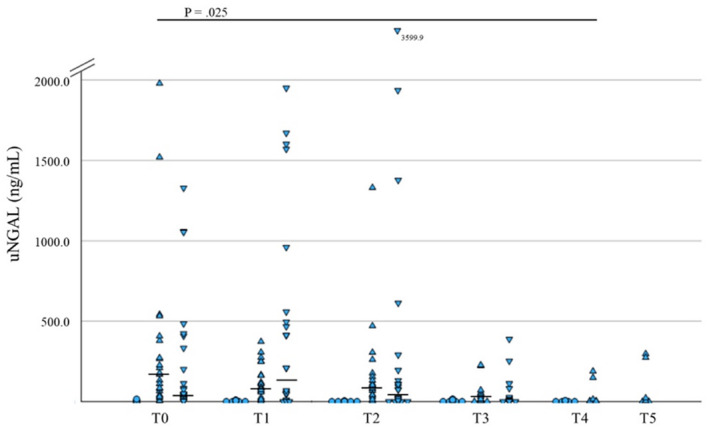
Changes in uNGAL over time: All data points of uNGAL in healthy (circles), AKI (triangles), and CI (inverted triangles) dogs. Variables were tracked from the initial presentation (T0) and followed up at 24 h (T1), 48 h (T2), 1 week (T3), 1 month (T4; only healthy and AKI), and 3 months (T5; only AKI). Median values are presented as a horizontal line if > 30 ng/mL.

In AKI dogs, sCr was significantly lower at T4 and T5 versus T0 (*p* = 0.01 and *p* = 0.02, respectively, *n* = 9), and sUrea and SDMA were significantly lower at T5 versus T0 (*p* = 0.05, and *p* = 0.01, respectively, both *n* = 9), whereas USG was significantly higher at T4 and T5 versus T2 (*p* = 0.02 and *p* = 0.01, respectively, *n* = 9) and UPC was significantly lower at T5 versus T2 (*p* = 0.04, *n* = 8), and uNGAL was significantly lower at T4 versus T0 (*p* = 0.02, *n* = 9, Figure 1).

**FIGURE 2:**
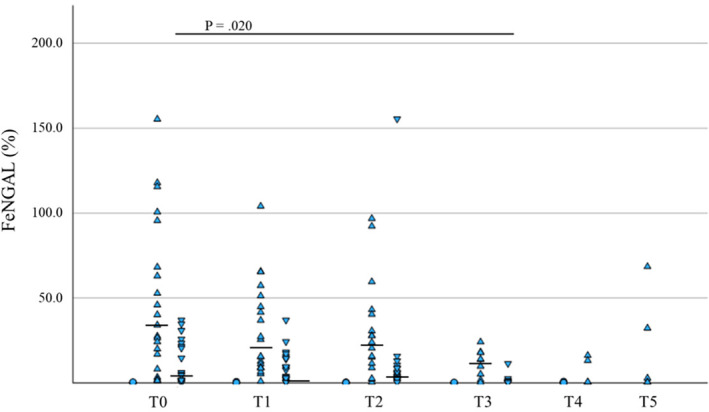
Changes in FeNGAL over time: All data points of FeNGAL in healthy (circles), AKI (triangles), and CI (inverted triangles) dogs. Variables were tracked from the initial presentation (T0) and followed up at 24 h (T1), 48 h (T2), 1 week (T3), 1 month (T4; only healthy and AKI), and 3 months (T5; only AKI). Median values are presented as a horizontal line if > 3.0%.

In CI dogs, sCrea was significantly lower at T1 and T2 versus T0 and at T3 versus T1 (*p* = 0.001, *p* = 0.002, and *p* = 0.04, respectively, *n* = 21), sUrea was significantly lower at T1 versus T0 (*p* = 0.01, *n* = 21), and UPC was significantly lower at T3 versus T0 and T1 (*p* = 0.01 and *p* < 0.001, respectively, *n* = 13), fractional excretion of NGAL (FeNGAL) was significantly lower at T3 versus T0 (*p* = 0.02, *n* = 5, Figure 2), and u/sNGAL significantly increased over time in CI dogs (*p* = 0.04, *n* = 10), but again no differences were found after multiple comparison tests.

### 3.3 Prediction of Survival in AKI and CI Dogs

Of the 24 dogs with AKI, 10 dogs survived (41.7%) until the last follow‐up 3 months later (T5). Of the 28 CI dogs, 20 survived (71.4%) until the last follow‐up 1 week after the initial presentation (T3).

**FIGURE 3:**
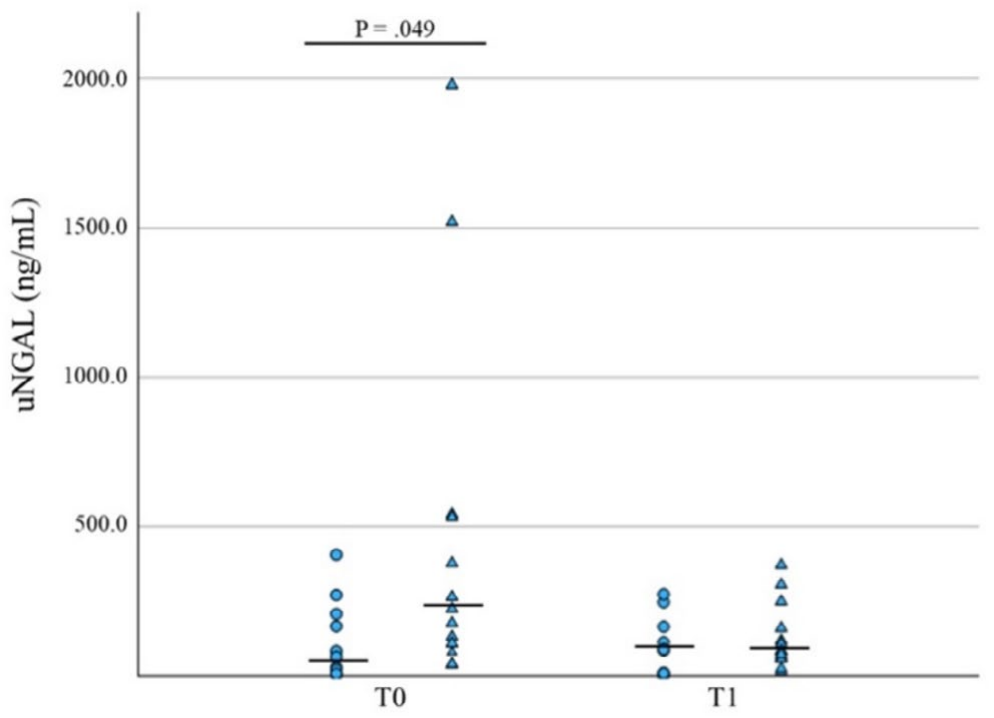
Comparative analysis of uNGAL concentrations at T0 and T1 in survivors (circles) versus nonsurvivors (triangles) in AKI dogs. Median values are presented as a horizontal line.

**FIGURE 4:**
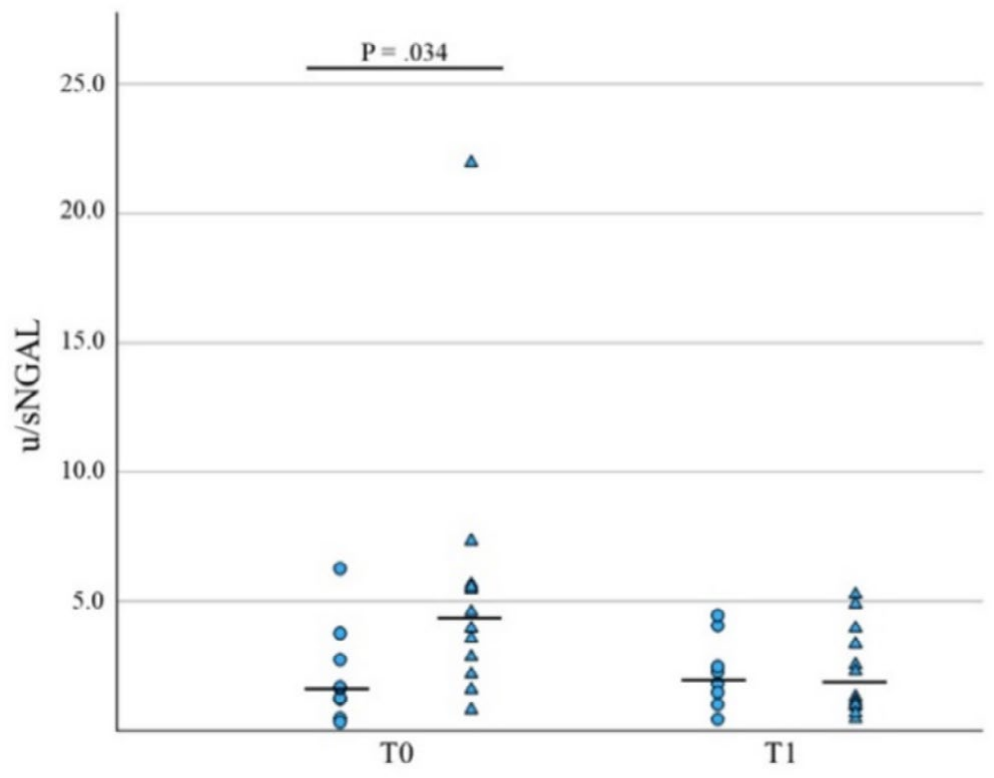
Comparative analysis of u/sNGAL levels concentrations at T0 and T1 in survivors (circles) versus nonsurvivors (triangles) in AKI dogs. Median values are presented as a horizontal line.

Dogs with AKI that did not survive had a significantly higher concentration of uNGAL and u/sNGAL ratio at T0 versus survivors (*p* = 0.05, *n* = 23, and *p* = 0.03, *n* = 21, respectively, Table 2, Figures 3 and 4). At T1, no significant differences were found between survivors and nonsurvivors.

**TABLE 2:** Median (range) of clinicopathological data, uTIMP‐2 and NGAL with absolute and normalized values at initial presentation (T0) of dogs with AKI and CI dogs that did and did not survive, CI dogs that did and did not develop AKI and AKI dogs that did and did not develop CKD.

Variable (unit) [reference interval]	AKI dogs		CI dogs		CI dogs		AKI dogs	
	Survivors	Nonsurvivors	Survivors	Nonsurvivors	No AKI	AKI	No CKD	CKD
sCr (mg/dL) [0.5–1.8]	4.8	3.7	0.8	0.8	0.9	0.6	4.3	5.3
	(2.3–13.2)	(1.4–17.6)	(0.3–3.7)	(0.4–2.3)	(0.3–3.7)	(0.5–0.8)	(2.3–13.2)	(2.4–12.3)
	*n* = 10	*n* = 14	*n* = 21	*n* = 7	*n* = 24	*n* = 4	*n* = 4	*n* = 6
sUrea (mmol/L) [3.2–10.3]	34.9	25.5	6.1	4.3	6.6	4.5	48.1	32.8
	(12.8–95.1)	(5.3–98.3)	(2.5–21.0)	(1.9–33.8)	(1.9–33.8)	(2.8–6.8)	(17.1–95.1)	(12.8–74.8)
	*n* = 10	*n* = 14	*n* = 21	*n* = 7	*n* = 24	*n* = 4	*n* = 4	*n* = 6
SDMA (μg/dL) [0.0–14.0]	32.5	38.0	13.0	11.0	13.5	9.5	18.0	32.5
	(12.0–> 100.0)	(14.0–> 100.0)	(6.0–29.0)	(6.0–46.0)	(6.0–46.0)^a^	(8.0–10.0)^a^	(12.0–52.0)	(20.0–86.0)
	*n* = 10	*n* = 13	*n* = 21	*n* = 7	*n* = 24	*n* = 4	*n* = 4	*n* = 6
USG [1.015–1.045]	1.013	1.015	1.026	1.028	1.028	1.021	1.014	1.012
	(1.008–1.016)	(1.005–1.024)	(1.006–1.050)	(1.004–1.039)	(1.006–1.050)	(1.004–1.026)	(1.008–1.014)	(1.008–1.016)
	*n* = 10	*n* = 14	*n* = 18	*n* = 7	*n* = 21	*n* = 4	*n* = 4	*n* = 6
UPC [< 0.50]	0.83	1.19	0.36	0.69	0.41	0.21	0.47	0.97
	(0.17–2.77)	(0.25–70.36)	(0.10–3.53)	(< 0.10–4.75)	(< 0.10–4.75)	(0.11–0.71)	(0.25–1.99)	(0.17–2.77)
	*n* = 10	*n* = 14	*n* = 18	*n* = 7	*n* = 21	*n* = 4	*n* = 4	*n* = 6
sNGAL (ng/mL)	45.6	68.7	18.2	64.9	29.9	25.2	13.5	50.6
	(5.2–69.1)	(14.9–269.5)	(3.3–108.0)^b^	(13.1–138.5)^b^	(3.3–138.5)	(13.9–44.2)	(5.2–61.0)	(23.9–69.1)
	*n* = 9	*n* = 12	*n* = 19	*n* = 7	*n* = 22	*n* = 4	*n* = 3	*n* = 6
uNGAL (ng/mL)	75.1	226.2	34.7	156.5	48.7	38.9	88.2	75.1
	(4.5–407.0)^b^	(39.5–1979.0)^b^	(< LOD–1058.4)	(48.7–1328.4)	(< LOD–1328.4)	(2.0–112.2)	(4.5–272.0)	(22.6–407.0)
	*n* = 10	*n* = 13	*n* = 19	*n* = 6	*n* = 21	*n* = 4	*n* = 4	*n* = 6
u_norm_NGAL	268.7	539.1	42.2	402.7	163.4	42.2	241.9	268.7
	(5.9–697.4)	(26.7–4602.3)	(< 0.1–1838.3)	(63.2–1953.5)	(< 0.1–1953.5)	(0.9–561.2)	(5.9–697.4)	(23.3–502.5)
	*n* = 10	*n* = 13	*n* = 16	*n* = 6	*n* = 18	*n* = 4	*n* = 4	*n* = 6
u/sNGAL (ng/mL)	1.6	4.3	3.9	2.0	3.9	1.7	1.6	1.5
	(0.3–6.3)	(0.8–22.0)	(< 0.1–29.7)	(1.0–20.5)	(< 0.1–29.7)	(0.1–2.5)	(0.3–2.8)	(0.5–6.3)
	*n* = 9	*n* = 12	*n* = 16	*n* = 6	*n* = 18	*n* = 4	*n* = 3	*n* = 6
u_norm_/sNGAL	48.9	80.8	79.6	69.4	106.1	18.6	33.3	50.1
	(4.4–77.4)^b^	(12.6–267.8)^b^	(0.2–419.7)	(11.1–301.2)	(0.2–419.7)	(0.6–127.0)	(4.4–76.4)	(5.1–77.4)
	*n* = 9	*n* = 12	*n* = 14	*n* = 6	*n* = 16	*n* = 4	*n* = 3	*n* = 6
FeNGAL (%)	20.0	49.3	4.7	3.8	9.8	1.1	8.1	23.2
	(1.0–100.6)	(2.0–155.3)	(< 0.1–37.0)	(0.5–34.7)	(< 0.1–37.0)	(< 0.1–6.0)	(1.0–100.6)	(1.2–62.9)
	*n* = 9	*n* = 12	*n* = 14	*n* = 6	*n* = 16	*n* = 4	*n* = 3	*n* = 6
uTIMP‐2 (pg/mL)	67.2	102.2	40.8	154.5	43.9	40.7	35.0	98.0
	(< 5.1–245.6)	(< 5.1–2132.0)	(< 5.1–484.6)	(< 5.1–700.0)	(< 5.1–700.0)	(< 5.1–296.2)	(< 5.1–152.7)	(40.5–245.6)
	*n* = 10	*n* = 14	*n* = 21	*n* = 7	*n* = 24	*n* = 4	*n* = 4	*n* = 6
u_norm_TIMP‐2 × 10^−8^	16.3	26.9	2.3	16.3	2.9	4.8	8.8	16.7
	(0.7–98.2)	(0.4–852.8)	(0.2–48.5)^b^	(0.7–76.6)^b^	(0.2–76.6)	(0.2–37.0)	(0.7–42.4)	(4.2–98.2)
	*n* = 10	*n* = 14	*n* = 18	*n* = 7	*n* = 21	*n* = 4	*n* = 4	*n* = 6

Abbreviations: AKI, acute kidney injury; CI, critically ill; FeNGAL, fractional excretion of NGAL; LOD, limit of detection NGAL: 0.6 pg/mL; sNGAL, serum neutrophil‐gelatinase associated lipocalin (NGAL); u/sNGAL, urinary to serum NGAL ratio; uNGAL, urinary NGAL; u_norm_/sNGAL, u/sNGAL with u_norm_NGAL; u_norm_NGAL, uNGAL normalized to urinary creatinine; u_norm_TIMP‐2 normalized to urinary creatinine; uTIMP‐2, urinary tissue inhibitor of metalloproteinase‐2.

^a^ Prediction of development of AKI.

^b^ Prediction of survival.

**FIGURE 5:**
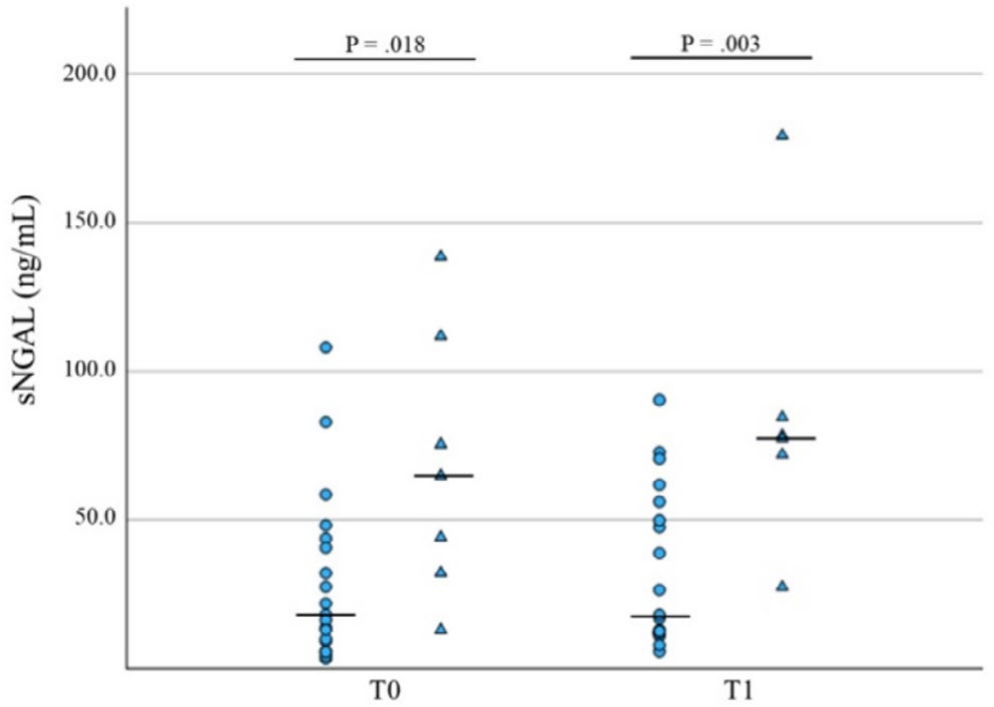
Comparative analysis of sNGAL levels concentrations at T0 and T1 in survivors (circles) versus nonsurvivors (triangles) in CI dogs. Median values are presented as a horizontal line.

**FIGURE 6:**
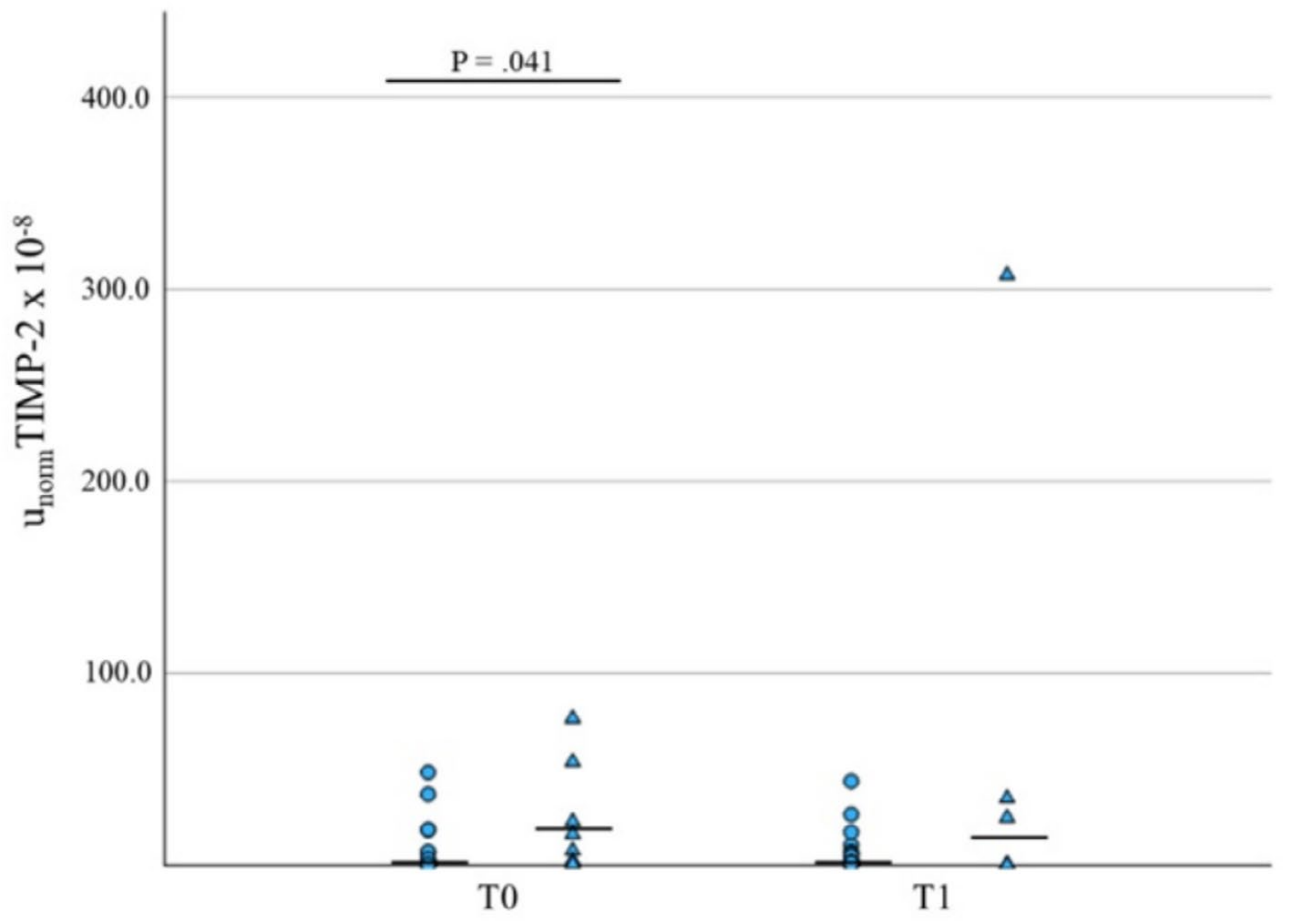
Comparative analysis of u_norm_TIMP‐2 levels concentrations at T0 and T1 in survivors (circles) versus nonsurvivors (triangles) in AKI dogs. Median values are presented as a horizontal line.

In CI dogs, sNGAL was significantly higher in non‐survivors at both T0 and T1 versus survivors (*p* = 0.02, *n* = 26, and *p* = 0.003, *n* = 26, respectively, Tables 2 and 3, Figure 5). At T0, u_norm_TIMP‐2 was also higher in CI dogs that did not survive versus survivors (*p* = 0.04, *n* = 25, Table 2, Figure 6).

**TABLE 3:** Median (range) of clinicopathological data, uTIMP‐2, and NGAL with absolute and normalized values 24 h after initial presentation (T1) of dogs with AKI and CI dogs that did and did not survive, CI dogs that did and did not develop AKI and AKI dogs that did and did not develop CKD.

Variable (unit) [reference interval]	AKI dogs		CI dogs		CI dogs		AKI dogs	
	Survivors	Nonsurvivors	Survivors	Nonsurvivors	No AKI	AKI	No CKD	CKD
sCr (mg/dL) [0.5–1.8]	3.7	3.9	0.6	0.9	0.6	0.6	3.0	3.7
	(1.8–12.2)	(0.7–15.7)	(0.3–1.1)	(0.3–1.5)	(0.3–1.5)	(0.6–0.9)	(1.8–12.2)	(2.1–12.1)
	*n* = 10	*n* = 14	*n* = 21	*n* = 6	*n* = 23	*n* = 4	*n* = 4	*n* = 6
sUrea (mmol/L) [3.2–10.3]	27.3	21.7	4.3	7.7	4.3	5.0	32.1	27.3
	(11.4–86.5)	(4.6–89.4)	(2.5–7.8)	(2.1–18.2)	(2.1–18.2)	(3.6–9.6)	(14.2–86.5)	(11.4–57.7)
	*n* = 10	*n* = 14	*n* = 21	*n* = 6	*n* = 23	*n* = 4	*n* = 4	*n* = 6
SDMA (μg/dL) [0–14]	30.0	38.0	12.0	15.5	13.0	11.0	33.5	30.0
	(11.0–99.0)	(8.0–89.0)	(6.0–25.0)	(7.0–48.0)	(6.0–48.0)	(10.0–30.0)	(11.0–99.0)	(21.0–90.0)
	*n* = 10	*n* = 13	*n* = 21	*n* = 6	*n* = 23	*n* = 4	*n* = 4	*n* = 6
USG [1.015–1.045]	1.010	1.011	1.014	1.024	1.014	1.025	1.010	1.012
	(1.005–1.016)	(1.005–1.019)	(1.001–1.050)	(1.009–1.049)	(1.001–1.050)	(1.025–1.030)	(1.005–1.011)	(1.008–1.016)
	*n* = 9	*n* = 14	*n* = 17	*n* = 6	*n* = 20	*n* = 3	*n* = 4	*n* = 5
UPC [< 0.50]	0.55	0.94	0.43	0.86	0.56	0.24	0.34	1.47
	(0.14–1.87)	(0.17–11.94)	(0.10–1.97)	(0.37–3.29)	(0.20–3.29)	(0.10–0.89)	(0.33–0.48)	(0.14–1.87)
	*n* = 8	*n* = 14	*n* = 18	*n* = 6	*n* = 21	*n* = 3	*n* = 3	*n* = 5
sNGAL (ng/mL)	61.1	73.6	17.5	77.9	43.2	21.7	31.7	61.5
	(5.4–195.5)	(< LOD–173.0)	(5.8–90.3)^a^	(27.5–179.2)^a^	(5.8–179.2)	(11.9–78.4)	(5.4–195.5)	(11.9–73.1)
	*n* = 9	*n* = 14	*n* = 20	*n* = 6	*n* = 22	*n* = 4	*n* = 4	*n* = 5
uNGAL (ng/mL)	91.1	87.0	55.2	686.5	208.0	60.5	48.6	166.1
	(< LOD–274.6)	(15.9–372.6)	(< LOD–1670.4)	(10.9–1950.0)	(< LOD–1950.0)	(< LOD–1570.2)	(< LOD–114.4)	(12.2–274.6)
	*n* = 9	*n* = 13	*n* = 18	*n* = 6	*n* = 21	*n* = 3	*n* = 4	*n* = 5
u_norm_NGAL	293.4	341.6	207.1	1345.0	346.2	104.3	312.2	274.6
	(24.8–506.1)	(31.2–1242.0)	(< 0.1–2141.5)	(54.7–2909.1)	(4.8–2909.1)	(< 0.1–1287.1)	(46.9–394.4)	(24.8–506.1)
	*n* = 8	*n* = 13	*n* = 15	*n* = 6	*n* = 18	*n* = 3	*n* = 3	*n* = 5
u/sNGAL (mL/ng)	2.0	1.8	4.2	11.1	5.5	11.2	1.8	2.3
	(0.4–4.5)	(0.5–5.3)	(< 0.1–41.3)	(0.2–20.0)	(< 0.1–41.3)	(2.3–20.0)	(0.4–2.5)	(1.0–4.5)
	*n* = 8	*n* = 12	*n* = 16	*n* = 6	*n* = 20	*n* = 2	*n* = 3	*n* = 5
u_norm_/sNGAL	47.0	59.6	151.9	133.6	151.9	101.8	85.6	44.6
	(16.0–87.0)	(13.4–132.0)	(3.4–420.9)	(7.6–344.2)	(3.4–420.9)	(39.4–164.1)	(16.0–87.0)	(20.8–82.9)
	*n* = 8	*n* = 12	*n* = 14	*n* = 6	*n* = 18	*n* = 2	*n* = 3	*n* = 5
FeNGAL (%)	13.2	31.2	8.4	8.5	8.4	8.9	15.3	11.2
	(6.5–104.0)	(0.9–65.6)	(0.2–24.3)	(1.1–36.9)	(0.2–36.9)	(2.3–15.4)	(6.5–104.0)	(8.7–51.2)
	*n* = 8	*n* = 12	*n* = 14	*n* = 6	*n* = 18	*n* = 2	*n* = 3	*n* = 5
uTIMP‐2 (pg/mL)	56.9	56.7	23.6	31.1	43.8	< 5.1	29.4	70.5
	(< 5.1–230.2)	(< 5.1–1236.2)	(< 5.1–74.5)	(< 5.1–1014.4)	(< 5.1–1014.4)	(< 5.1–60.9)	(< 5.1–69.1)	(40.1–230.2)
	*n* = 9	*n* = 13	*n* = 20	*n* = 6	*n* = 22	*n* = 4	*n* = 4	*n* = 5
u_norm_TIMP‐2 × 10^−8^	19.7	18.9	2.3	12.6	2.8	0.7	19.2	20.1
	(2.4–47.0)	(1.0–450.4)	(0.4–43.8)	(0.3–307.4)	(0.3–307.4)	(0.4–10.5)	(2.4–23.8)	(4.0–47.0)
	*n* = 8	*n* = 13	*n* = 16	*n* = 6	*n* = 19	*n* = 3	*n* = 3	*n* = 5

Abbreviations: AKI, acute kidney injury; CI, critically ill; FeNGAL, fractional excretion of NGAL; LOD, limit of detection NGAL: 0.6 pg/mL; sNGAL, serum neutrophil‐gelatinase associated lipocalin (NGAL); u/sNGAL, urinary to serum NGAL ratio; uNGAL, urinary NGAL; u_norm_/sNGAL, u/sNGAL with u_norm_NGAL; u_norm_NGAL, uNGAL normalized to urinary creatinine; u_norm_TIMP‐2 normalized to urinary creatinine; uTIMP‐2, urinary tissue inhibitor of metalloproteinase‐2.

^a^ Prediction of survival.

### 3.4 Prediction of the Development of Hospital‐Acquired AKI in CI Dogs

**FIGURE 7:**
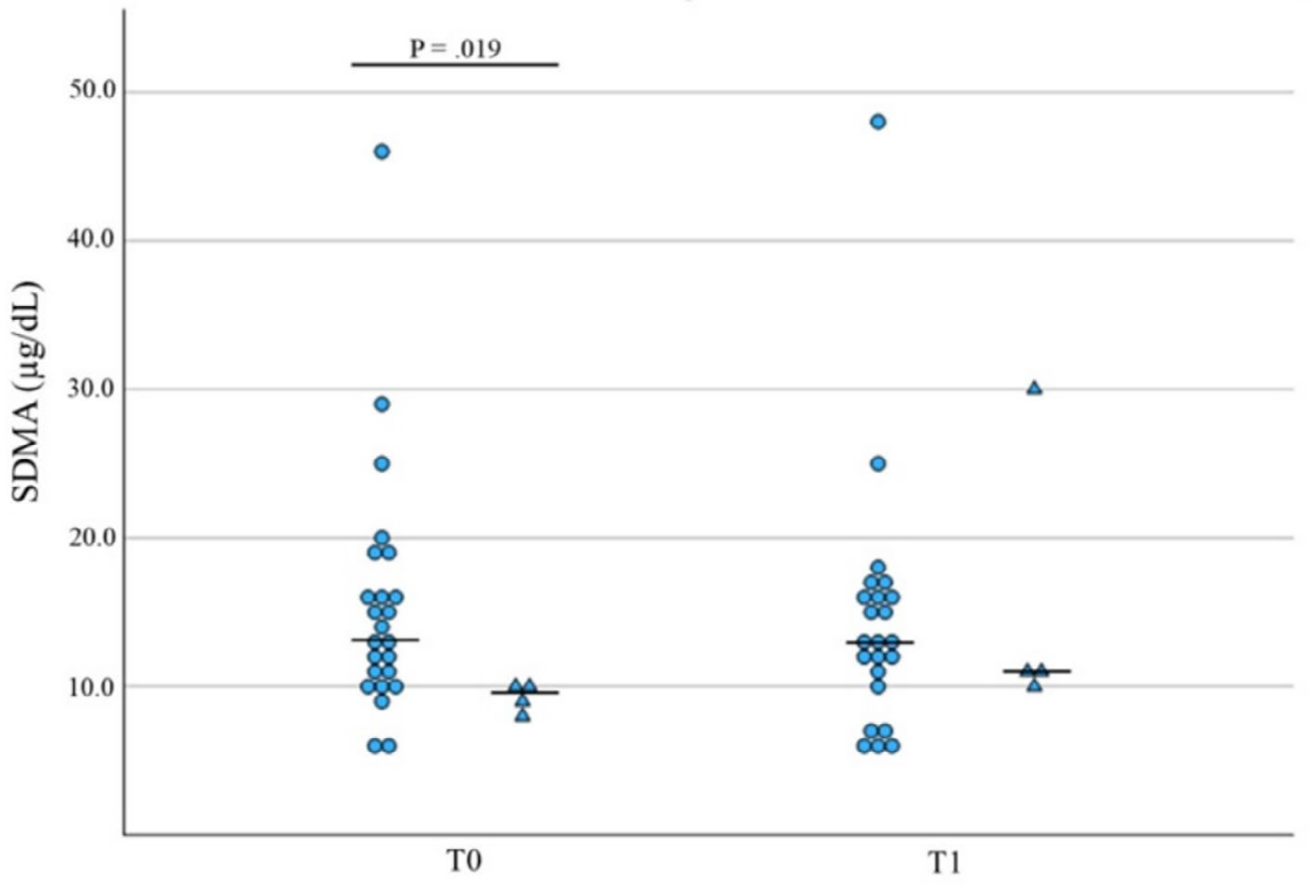
Box plot of T0 SDMA levels predicting AKI development in CI dogs. *p* ≤ 0.05 was considered significant. Comparative analysis of SDMA levels concentrations at T0 in dogs that did not develop AKI (circles) versus dogs that developed AKI (triangles) in CI dogs. Median values are presented as a horizontal line.

Of the 28 CI dogs, four developed AKI during hospitalization of which one died during the study period (14.3% and 3.6%, respectively). In those dogs that developed AKI, SDMA concentrations were lower at T0 versus dogs that did not develop AKI (*p* = 0.02, *n* = 28; Table 2, Figure 7).

### 3.5 Prediction of CKD in AKI Dogs

Development of CKD occurred in six dogs that initially were presented with AKI (25%). According to IRIS staging for CKD, these dogs were staged as CKD stage 1, normotensive, non‐proteinuric; CKD stage 2, normotensive, non‐proteinuric; CKD stage 2 normotensive, borderline proteinuric; CKD stage 2 hypertensive, non‐proteinuric; CKD stage 3, borderline hypertensive, proteinuric; and CKD stage 3 hypertensive, proteinuric, respectively, at 3 months after their initial presentation.

No significant differences were found at T0 and T1 between dogs that did versus those that did not develop CKD (Tables 2 and 3).

## 4 Discussion

Our prospective study aimed to identify prognostic biomarkers for predicting survival and disease progression in AKI and CI dogs. We found that u_norm_TIMP‐2 measured at T0 and sNGAL measured at T0 and T1 could predict survival in CI dogs. A significant difference in SDMA concentrations was found at T0 between CI dogs that developed AKI and those that did not, but given the considerable overlap between the two groups, the clinical relevance of this finding was considered questionable. No predictive markers for the development of CKD in dogs with AKI were identified.

The most important finding in our study was that CI dogs that did not survive had higher u_norm_TIMP‐2 and sNGAL concentrations at admission and higher sNGAL at admission. More detailed prognostic information could assist owners and veterinarians in decision‐making about whether to pursue intensive care hospitalization or consider euthanasia.

Neutrophil gelatinase‐associated lipocalin is generated in the bone marrow during the process of myelopoiesis and held within the gelatinase granules of neutrophils [22]. Additionally, it can be expressed in response to inflammatory mediators in various non‐hematopoietic tissues, including the kidney, colon, trachea, and lung epithelium [23]. Given the source of NGAL and the inflammatory state at the time of admission, increased NGAL concentrations in CI dogs are expected. Previous studies in CI dogs with gastric dilatation volvulus found that plasma NGAL and uNGAL remained increased up to 48 h post‐surgery [24]. Another study found that sNGAL concentrations were increased until 48 h post‐surgery in dogs with sepsis that required emergency laparotomy [25].

The more recent biomarker TIMP‐2 is an effective indicator of cell cycle arrest and indicates the prognosis of AKI in humans [26]. It is a key regulator of extracellular matrix turnover and is involved in numerous physiological and pathological processes, particularly in kidney injury and repair [27]. The therapeutic utility of cell cycle arrest biomarkers such as uTIMP‐2 and insulin‐like growth factor‐binding protein 7 (IGFBP7) for risk stratification of AKI in CI people was evaluated in a large prospective multicenter trial. Increased cell cycle arrest biomarker concentrations were associated with a higher risk of developing AKI and worse overall outcomes, including a higher likelihood of requiring renal replacement therapy and increased mortality rate [28]. Another study in humans aimed to determine if serial measurements of [TIMP‐2] × [IGFBP7] could predict AKI during the first 7 days of critical illness. The study found that these measurements, taken at baseline, 12 and 24 h, and up to 3 days, are effective in the prediction of AKI [29]. We did not find that TIMP‐2 was predictive for the development of AKI, which might be related to the small number of CI dogs that developed AKI. However, u_norm_TIMP‐2 was predictive of the outcome (survival) of CI dogs.

In critical illness and sepsis, AKI is recognized as a major factor contributing to morbidity and mortality in people. Even a mild increase in sCr concentration during hospitalization increases the risk of in‐hospital death [30, 31]. Progressive acute azotemia has been shown to predict mortality in dogs and cats [32]. The short‐term prognosis of AKI is influenced by several factors, including its underlying cause, concurrent health conditions, complications, and available treatment options [33, 34]. Early detection of AKI in hospitalized dogs is essential for improving their prognosis, because it allows for timely intervention, which can markedly enhance outcomes. Urinary biomarkers offer a promising approach for achieving early AKI detection, providing veterinarians with tools to identify kidney stress at an early stage before clinical signs become apparent. This proactive approach is key to optimizing treatment and ultimately survival and recovery of affected dogs [35]. Previous studies have shown that sNGAL can predict development of AKI in CI people [36]. However, data considering the prediction of AKI in CI dogs is lacking. Unexpectedly, in our study, SDMA concentrations at baseline were higher in CI dogs that did not develop AKI compared with CI dogs that did develop AKI. Although there was considerable overlap between both outcome groups and type 1 statistical error cannot be ruled out, this finding does raise questions about the clinical relevance of SDMA in this situation. Potential explanations of the higher baseline SDMA concentrations in CI dogs that developed AKI could be the small number of CI dogs that developed AKI in our study or nonrenal influences on serum SDMA concentration. Also, previous studies found conflicting results: one prospective study found that SDMA could correctly identify dogs with AKI (*n* = 48) and dogs with CKD (*n* = 29) compared with healthy dogs (*n* = 18), but could not differentiate between the renal diseases [37]. However, in another prospective study on CI dogs, no difference in SDMA was found between severely affected dogs (*n* = 12) compared to those with mild to moderate disease (*n* = 10), between CI dogs (*n* = 22) and healthy dogs (*n* = 7), and between survivors (*n* = 18) and nonsurvivors (*n* = 4) [38]. In general, a comprehensive approach to AKI diagnosis and prognosis in human medicine was suggested by combining multiple biomarkers. For example, integrating NGAL and interleukin‐18 (IL‐18) with clinical variables improves the prediction of adverse outcomes [39] and this approach also might be recommended in veterinary medicine.

Acute kidney injury is common in veterinary practice and is associated with high morbidity and mortality. Dogs with AKI often require prolonged hospitalization, which is associated with substantial financial investment, and are at risk of developing CKD [3]. Understanding the long‐term prognosis of AKI is essential for veterinary clinicians and pet owners, because it directly impacts clinical decision‐making and management strategies. Additionally, comprehensive data on long‐term outcomes will allow clinicians to develop effective monitoring protocols and customize therapeutic interventions for dogs recovering from AKI. Previous studies have developed a scoring system to help predict outcomes in dogs with AKI that require hemodialysis. This system is based on the clinical signs and clinicopathological abnormalities observed on the first day of hospitalization [33]. Other studies have suggested that urinary biomarkers such as γ‐glutamyl transpeptidase, heat‐shock protein 70, and interleukin 6 might predict AKI in hospitalized dogs [40]. Urinary NGAL is a sensitive marker for AKI in dogs, but its specificity is influenced by systemic inflammation. A sensitivity of 85% [41] and 91% [42] and specificity of 70% [41] and 81% [42] at diagnosing AKI in dogs based on uNGAL were reported. High NGAL concentrations observed in an earlier study in both intrinsic AKI and volume‐responsive AKI also indicate tubular damage in cases of transient AKI [43]. However, we did not find significant predictive value for the examined biomarkers for survival in dogs with AKI. At admission, USG was significantly lower in dogs with AKI compared with healthy and CI dogs, which is not unexpected because most dogs had advanced AKI. During hospitalization, USG was mostly in the isosthenuric range both in the AKI group and in the CI group, which can be largely explained by fluid therapy. However, the USG stays in the isosthenuric range in most AKI dogs at the 1‐month follow‐up visit and becomes more concentrated at the 3‐month follow‐up visit, indicating that several months are needed for kidney recovery. This finding aligns with a study of humans suggesting that recovery after AKI may take several months [44].

In people, a history of AKI is linked to a higher risk of developing CKD [45]. Recent findings in dogs and cats indicate that both conditions are closely related and that animals with CKD also have ongoing active damage [46]. A study on hospitalized people with AKI demonstrated an association between uNGAL concentrations and persistent AKI, as well as major adverse kidney events at 30 and 365 days [47]. No biomarker predictive for CKD development in dogs with AKI could be identified in our study.

Recently, an assay for the tubular biomarker urinary cystatin B was commercialized to assess for active kidney damage in dogs and cats. Its use in clinics is mainly to identify small animals at risk for AKI [48, 49] and to differentiate stable from progressive CKD [50]. It would have been very interesting to assess urinary cystatin B in the dogs of our study to further enhance understanding of AKI, but the test was not yet available at the time of study design and sample collection.

Our study had some limitations. The primary limitation was the small sample size, particularly during the follow‐up period. Furthermore, not all dogs provided enough samples for analysis at every time point, and many NGAL samples were either below the LOD or too high, resulting in limited data availability. This may have decreased the study's statistical power.

Although the overall group sizes were adequate, the subgroups (such as those developing AKI or non‐survivors) were small, making it difficult to draw definitive conclusions. As a result, it was not possible to establish accurate cut‐offs for the biomarkers. Additionally, CI dogs, both upon admission and during hospitalization, are at risk for hospital‐acquired AKI, and it was challenging to entirely rule out AKI IRIS grade 1 in these cases. Also, most dogs included with AKI had IRIS grade 2 or higher because diagnosing IRIS grade 1 remains difficult. However, it is anticipated that stress and kidney damage biomarkers would be particularly valuable in detecting AKI at grade 1. Samples were stored at −80°C for up to 2 years before further analysis. Data in people show that NGAL concentrations remain stable when urine samples are stored at −80°C. One study showed stable NGAL concentrations for at least 6 months [51] whereas a study in children showed that after 3 years of storage, urinary NGAL concentrations decreased by just 0.42%, and after 5 years, the decrease was 0.84%. These findings support the feasibility of long‐term urine storage for up to 5 years with minimal degradation of NGAL [52]. Another study investigated the stability of two renal tubular stress biomarkers, TIMP‐2 and IGFBP‐7, which are combined in the NephroCheck test to calculate an AKI risk score. The findings indicated that the AKI risk score remained relatively stable when samples were stored at −80°C for up to 8 weeks [53]. However, studies on the stability of TIMP‐2 for long‐term storage over 2 years are lacking.

Concentrations of TIMP‐2 below the LOQ (20 pg/mL) were included in our analysis, but the performance of the ELISA kit for concentrations under 20 pg/mL needs further validation because many samples fall below the LOD, potentially impacting the accuracy of the linearity results. Importantly, no results were found in the equivocal zone between 5.1 and 20 pg/mL. Although we were unable to investigate this situation further, the good intra‐assay percentage coefficient of variation for low values lends confidence to the reliability of the data.

In conclusion, our study emphasizes the importance of assessing biomarkers for predicting outcomes in CI dogs. We found higher sNGAL measured at both T0 and T1 and u_norm_TIMP‐2 measured at T0 in hospitalized CI dogs that survived the hospitalization period. The value of SDMA at baseline to identify dogs at risk of developing AKI during hospitalization warrants further evaluation. Monitoring these biomarkers upon admission could enhance diagnostic accuracy, support clinical decision‐making, and improve early detection of hospital‐acquired AKI. These findings might have clinically relevant implications for the management and treatment of CI and AKI dogs in veterinary medicine, refining prognostic tools, offering potential pathways for early intervention improving clinical outcomes, and managing owner expectations.

## Disclosure

Authors declare no off‐label use of antimicrobials.

## Ethics Statement

Approval granted by the local ethical committee of the Faculty of Veterinary Medicine, Ghent University, Belgium, and the deontological committee of the Belgian Federal Agency for the Safety of the Food Chain (EC 2020/067, Deontological committee: 2018‐86). Authors declare human ethics approval was not needed for this study.

## Conflicts of Interest

The authors declare no conflicts of interest.

## Supporting information


Supplementary Table S1.
