# Supplementary material for: Outcome in Critically Ill Dogs and Dogs With Acute Kidney Injury Based on Neutrophil Gelatinase‐Associated Lipocalin and Tissue Inhibitor of Metalloproteinase‐2
Source: J Vet Intern Med. 2025 Feb 26;39(2):e70024. doi: 10.1111/jvim.70024 (PMC11863212; doi:10.1111/jvim.70024)
Supplement: Supplementary file 1 — Supplementary Table S1. [file JVIM-39-e70024-s001.docx]

**Supplementary Table 1.** Median (range) of assessed blood and urinary variables of the different study groups at different time points. Statistical changes between different groups at each time point are indicated.

|  | Healthy dogs | | | | | Dogs with AKI | | | | | | CI dogs | | | |
| --- | --- | --- | --- | --- | --- | --- | --- | --- | --- | --- | --- | --- | --- | --- | --- |
| Variable (unit)  [reference interval] | T0 | T1 | T2 | T3 | T4 | T0 | T1 | T2 | T3 | T4 | T5 | T0 | T1 | T2 | T3 |
| sCr (mg/dL) [.5-1.8] | 1.0  (.6-1.6)^b^  *n = 10* | .9  (.6-1.2)^b^  *n = 10* | .8  (.6-1.3)^b^  *n = 10* | .9  (.6-1.2)  *n=10* | .9  (.6-1.3)  *n=10* | 3.9 (1.4-17.6)^ac^  *n=24* | 3.8 (.7-15.7)^ac^  *n=24* | 2.2 (.6-15.8)^ac^  *n=21* | 1.8 (.6-5.1)^c^  *n=12* | 1.1 (.7-3.3)  *n=10* | 1.3 (.9-4.1)  *n=10* | .8 (.3-3.7)^b^  *n = 28* | .6 (.3-1.5)^b^  *n = 26* | .5 (.3-1.5)^b^  *n = 26* | .7 (.1-1.2)^b^  *n = 21* |
| sUrea (mmol/L)  [3.2-10.3] | 4.8 (2.1-11.8)^b^  *n=10* | 5.2 (2.1-13.9)^b^  *n=10* | 5.7 (2.8-7.8)^b^  *n=10* | 5.2 (3.9-8.9)  *n =10* | 4.8 (2.1-11.8)^b^  *n=10* | 25.5 (5.3-98.3)^ac^  *n=24* | 21.7 (4.6-89.4)^ac^  *n=24* | 15.0 (5.0-88.3)^ac^  *n=21* | 11.2 (3.2-66.9)^c^  *n=12* | 8.9 (5.7-25.6)^a^  *n=10* | 8.9 (3.9-36.3)  *n=10* | 6.1 (1.9-33.8)^b^  *n=28* | 4.3 (2.1-18.2)^b^  *n=27* | 4.3 (2.1-72.0)^b^  *n=26* | 5.0 (2.8-72.0)^b^  *n=21* |
| SDMA (µg/dL) [0-14] | 12.0 (7.0-14.0)^b^  *n=10* | 10.5 (7.0-13.0)^b^  *n=10* | 11.5 (6.0-13.0)^b^  *n=10* | 11.0 (6.0-15.0)^b^  *n=10* | 12.0 8.0-13.0)^b^  *n=10* | 37.0 (12.0->100.0)^ac^  *n=23* | 37.0 (8.0-99.0)^ac^  *n=23* | 27.0 (11.0-89.0)^ac^  *n=21* | 24.5 (12.0-75.0)^ac^  *n=12* | 17.0 (9.0-35.0)^a^  *n=10* | 16.5 (11.0-32.0)  *n=10* | 12.5 (6.0-46.0)^b^  *n=28* | 13.0 (6.0-48.0)^b^  *n=27* | 11.0 (6.0-21.0)^b^  *n=26* | 13.0 (8.0-19.0)^b^  *n=21* |
| USG  [1.015-1.045] | 1.035 (1.022-1.050)^b^  *n=10* | 1.045 (1.010->1.050)^bc^  *n=10* | 1.045 (1.030->1.050)^bc^  *n=10* | 1.040 (1.018-1.050)^b^  *n=9* | 1.046 (1.018->1.050)^b^  *n=10* | 1.014 (1.005-1.024)^ac^  *n=24* | 1.010 (1.005-1.019)^ac^  *n=23* | 1.010 (1.005-1.023)^a^  *n=20* | 1.012 (1.006-1.025)^ac^  *n=12* | 1.017 (1.011-1.050)^a^  *n=9* | 1.024 (1.011-1.050)  *n=10* | 1.026 (1.004-1.050)^b^  *n=25* | 1.014 (1.001-1.050)^ab^  *n=23* | 1.011 (1.006-1.045)^a^  *n=23* | 1.027 (1.009-1.045)^b^  *n=17* |
| Healthy dogs | | | | | | Dogs with AKI | | | | | | CI dogs | | | |
| Variable (unit)  [reference interval] | T0 | T1 | T2 | T3 | T4 | T0 | T1 | T2 | T3 | T4 | T5 | T0 | T1 | T2 | T3 |
| UPC  [< .50] | .07 (.05-.11)^bc^  *n=10* | .08 (.04-.14)^bc^  *n=10* | .07 (.04-.10)^bc^  *n=10* | .08 (.05-.27)^b^  *n=9* | .08 (.04-.14)^b^  *n=10* | .89 (.17-70.36)^a^  *n=24* | .80 (.14-11.94)^a^  *n=22* | .74 (.18-11.98)^a^  *n=20* | .55 (.07-5.80)^a^  *n=12* | .14 (.04-2.54)^a^  *n=9* | .11 (.04-2.48)  *n=10* | .40 (<.01-4.75)^a^  *n=25* | .53 (.10-3.29)^a^  *n=24* | .40 (.07-1.40)^a^  *n=23* | .13 (.05-3.71)  *n=17* |
| sNGAL  (ng/mL) | 8.4 (4.0-31.9)^bc^  *n=10* | 11.8 (4.9-29.1)^bc^  *n=10* | 7.2 (<LOD-25.6)^bc^  *n=10* | 9.8 (<LOD-28.8)^b^  *n=9* | 10.1 (5.6-26.9)  *n=10* | 57.2 (5.2-269.5)^a^  *n=21* | 61.5 (<LOD-195.5)^a^  *n=23* | 48.9 (<LOD-150.7)^a^  *n=21* | 52.5 (<LOD-182.8)^a^  *n=13* | 15.4 (LOD-77.7)  *n=9* | 12.6 (6.4-55.9)  *n=9* | 29.8 (3.3-138.5)^a^  *n=26* | 33.1 (5.8-179.2)^a^  *n=26* | 32.1 (<LOD-118.4)^a^  *n=25* | 22.0 (<LOD-75.8)  *n=18* |
| uNGAL  (ng/mL) | 1.1 (.1-15.3)^bc^  *n=9* | <LOD (<LOD-10.2)^bc^  *n=7* | <LOD (<LOD-4.8)^bc^  *n=9* | .6 (LOD-16.1)  *n=9* | 1.5 (<LOD-5.9)  *n=10* | 172.9 (4.5-1979.0)^a^  *n=23* | 89.3 (.6-372.6)^a^  *n=22* | 99.4 (<LOD-1330.8)^a^  *n=20* | 30.4 (<LOD-226.8)  *n=13* | 3.7 (2.7-188.6)  *n=9* | 4.1 (<LOD-297.0)  *n=10* | 45.1 (<LOD-1328.4)^a^  *n=25* | 138.5 (<LOD-1950.0)^a^  *n=24* | 73.0 (<LOD-3599.9)^a^  *n=25* | 3.3 (<LOD-388.0)  *n=20* |
| u_norm_NGAL | .4 (<.1-10.6)^bc^  *n=9* | <.1 (<.1-4.5)^bc^  *n=7* | <.1 (<.1-1.7)^bc^  *n=9* | .3 (<.1-6.8)^b^  *n=8* | .8 (<.1-4.9)  *n=10* | 338.3 (5.9-4602.3)^a^  *n=23* | 312.2 (24.8-1242.0)^a^  *n=21* | 450.8 (<.1-2138.2)^a^  *n=19* | 72.8 (<.1-492.9)^a^  *n=12* | 1.9 (.2-449.1)  *n=9* | 3.2 (<.1-651.9)  *n=10* | 119.8 (<.1-1953.5)^a^  *n=22* | 282.9 (<.1-2909.1)^a^  *n=21* | 161.4 (<.1-25713.6)^a^  *n=22* | 5.2 (<.1-206.5)  *n=16* |
| Healthy dogs | | | | | | Dogs with AKI | | | | | | CI dogs | | | |
| Variable (unit)  [reference interval] | T0 | T1 | T2 | T3 | T4 | T0 | T1 | T2 | T3 | T4 | T5 | T0 | T1 | T2 | T3 |
| u/s NGAL | .1 (<.1-.9)^bc^  *n=9* | .2 (.2-1.6)^c^  *n=3* | .2(.1-.9)^bc^  *n=4* | .3 (<.1-.6)  *n=4* | .2 (.1-.7)  *n=6* | 2.9 (.3-22.0)^a^  *n=21* | 2.0 (.5-5.3)  *n=20* | 2.1 (.1-11.1)^a^  *n=18* | 1.3 (.1-3.6)  *n=9* | .4 (.1-2.4)  *n=6* | .3 (.1-7.1)  *n=7* | 2.6 (<.1-29.7)^a^  *n=22* | 5.5 (.1-41.3)^a^  *n=22* | 2.2 (.1-74.1)^a^  *n=21* | .5 (<.1-8.2)  *n=14* |
| u_norm_/sNGAL (mL/ng) | .4 (<.1-4.5)^bc^  *n=9* | .8 (.6-7.2)^c^  *n=3* | .6 (.3-3.3)^bc^  *n=4* | 1.2 (.5-2.4)^b^  *n=4* | 1.0 (.6-6.6)  *n=6* | 62.2 (4.4-267.8)^a^  *n=21* | 50.9 (13.4-132.0)  *n=20* | 92.4 (2.8-209.1)^a^  *n=17* | 32.2 (2.3-79.5)^ac^  *n=9* | 3.8 (.8-57.8)  *n=6* | 3.0 (.5-169.1)  *n=7* | 79.6 (.2-419.7)^a^  *n=20* | 149.9 (3.4-420.9)^a^  *n=20* | 90.7 (3.3-5290.9)^a^  *n=19* | 5.6 (.2-168.0)^b^  *n=11* |
| FeNGAL (%) | <.1 (<.1-.5)^bc^  *n=9* | .1 (.1-.7)^b^  *n=3* | <.1 (<.1-.4)^b^  *n=4* | .1 (<.1-.3)^b^  *n=4* | .1 (<.1-.8)  *n=6* | 34.0 (1.0-155.3)^ac^  *n=21* | 20.5 (.9-104.0)^ac^  *n=20* | 23.9 (.4-96.8)^ac^  *n=17* | 13.7 (.1-24.1)^ac^  *n=9* | .4 (.1-16.1)  *n=6* | .5 (.1-68.4)  *n=7* | 4.7 (<.1-37.0)^ab^  *n=20* | 8.4 (.2-37.0)^b^  *n=20* | 4.0 (.3-155.5)^b^  *n=19* | .2 (<.1-11.4)^b^  *n=11* |
| uTIMP-2  (pg/mL) | 27.6 (<5.1-2630.0)  *n=10* | <5.1 (<5.1-47.2)^b^  *n=10* | <5.1 (<5.1-47.2)^b^  *n=10* | 23.0 (<5.1-69.4)  *n=10* | <5.1 (<5.1-<5.1)  *n=8* | 76.0 (<5.1-2132.0)  *n=24* | 56.8 (<5.1-1236.2)^a^  *n=22* | 52.3 (<5.1-147.9)^a^  *n=21* | <5.1 (<5.1-107.4)  *n=12* | <5.1 (<5.1-765.6)  *n=9* | 33.5 (<5.1-499.0)  *n=8* | 43.9 (<5.1-700.0)  *n=28* | 23.7 (<5.1-1014.4)  *n=26* | <5.1 (<5.1-83.6)  *n=25* | 25.5 (<5.1-66.2)  *n=18* |
| u_norm_TIMP-2 x10^-8^ | 1.5 (.2-74.7)^b^  *n=10* | .2 (.1-4.0)^bc^  *n=10* | .2 (.1-1.9)^bc^  *n=10* | .6 (.1-2.7)^b^  *n=9* | .3 (.2-.6)^b^  *n=8* | 18.1 (.4-852.8)^ac^  *n=24* | 19.2 (1.0-450.4)^a^  *n=21* | 9.2 (1.0-61.6)^a^  *n=20* | 2.9 (.5-23.9)^a^  *n=12* | .5 (.2-107.8)^a^  *n=9* | 3.0 (.2-118.8)  *n=8* | 2.9 (.2-76.6)^b^  *n=25* | 2.3 (.4-307.4)^a^  *n=22* | 3.5 (.4-40.2)^a^  *n=22* | 1.2 (.2-23.0)  *n=14* |

^a^ significant when Healthy compared to AKI

^b^ significant when Healthy compared to CI

^c^ significant when AKI compared to CI

**Abbreviations:** AKI, acute kidney injury; CI, critically ill; sNGAL, serum neutrophil-gelatinase associated lipocalin (NGAL); uNGAL, urinary NGAL; u_norm_NGAL, uNGAL normalized to urinary creatinine; u/sNGAL, urinary to serum NGAL ratio; u_norm_/sNGAL, u/sNGAL with u_norm_NGAL; FeNGAL, fractional excretion of NGAL; uTIMP-2, urinary tissue inhibitor of metalloproteinase-2; u_norm_TIMP-2 normalized to urinary creatinine; LOD: limit of detection NGAL: .6 pg/mL.
